# Intrahippocampal injection in mice used for experimental studies in Alzheimer’s disease: a challenging procedure for neuroscience purposes

**DOI:** 10.25122/jml-2026-0017

**Published:** 2026-02

**Authors:** Alexandru Laslo, Laura Laslo, Klara Brinzaniuc

**Affiliations:** 1 Department of Urology, George Emil Palade University of Medicine, Pharmacy, Science, and Technology of Targu Mures, Targu Mures, Romania; 2 Doctoral School of Medicine and Pharmacy, George Emil Palade University of Medicine, Pharmacy, Science and Technology of Targu Mures, Targu Mures, Romania; 3 Faculty of Medicine, George Emil Palade University of Medicine, Pharmacy, Science, and Technology of Targu Mures, Targu Mures, Romania; 4 Department of Anatomy, George Emil Palade University of Medicine, Pharmacy, Science, and Technology of Targu Mures, Targu Mures, Romania

**Keywords:** intrahippocampal injection, neuroscience, experiment

## Abstract

Neuroscience has advanced over the years largely due to animal experiments, particularly in mice. These experiments are generally challenging and require thorough preparation to be successfully carried out. The training required to perform procedures on mice must be rigorous to minimize the risk of errors that could lead to experimental failure and, equally important, to prevent unnecessary suffering of the animals involved. In this study, we present a detailed description of the surgical procedure for intrahippocampal injection in mice using a motorized stereotaxic system equipped with synchronized drilling and microinjection modules. The protocol emphasizes precise anatomical targeting, controlled infusion parameters, and standardized procedural steps designed to enhance reproducibility and minimize tissue trauma. Key aspects of the technique include stereotaxic atlas alignment, skull reference acquisition, controlled drilling to the dura mater, and microinjection of small tracer volumes under physiologically relevant conditions. This methodological framework provides a reliable platform for investigating brain parenchymal transport mechanisms, including intramural periarterial drainage pathways implicated in neurodegenerative disorders such as Alzheimer’s disease.

## Introduction

Although Alzheimer’s disease (AD) was first identified over 119 years ago, its underlying pathophysiology remains incompletely elucidated. Nevertheless, advancements in medical science and the continuous refinement of research methodologies have led to the identification of both macroscopic and microscopic markers, providing enhanced insights into the mechanisms driving the onset and progression of the disorder [1].

The earliest accounts of anatomical and pathomorphological alterations associated with Alzheimer’s disease were documented by Alois Alzheimer in 1906. Postmortem analyses of patients’ brains revealed macroscopic abnormalities that are now integral to the diagnostic framework for definitive AD [1]. Autopsy findings conducted by forensic pathologists confirm a definitive diagnosis in approximately 85% of cases assessed in medico-legal institutions globally.

Atrophy of the hippocampus and cerebral cortex observed in affected brains is attributed to progressive neuronal loss in these regions, an effect that intensifies with advancing age and disease stage [2,3]. This neurodegeneration contributes significantly to cognitive decline, particularly due to hippocampal involvement, which is further linked to Tau protein aggregation. Structural brain alterations are most pronounced in the amygdala, cingulate cortex, and associative cortical areas of the frontal, temporal, and parietal lobes, as well as in certain subcortical nuclei [1,4].

Microscopic examinations reveal hallmark pathological features of AD, including the accumulation of amyloid plaques and protein fragments. These deposits follow a predictable anatomical trajectory, initiating in the pre-olfactory cortex and progressively involving the entorhinal cortex, the Cornu Ammonis 1 (CA1) sector of the hippocampus, and ultimately the associative cortices of the frontal, parietal, and temporal lobes [5]. The distribution and density of these plaques have been shown to correlate closely with the clinical severity of dementia. These pathological processes also trigger an immune response, characterized by the activation of central nervous system phagocytes, including monocytes and resident macrophages [4].

In a healthy organism, β-amyloid exists as a small, water-soluble peptide. It is generated through the proteolytic cleavage of the native amyloid precursor protein (APP) by the sequential actions of α-secretase, β-secretase, and γ-secretase [6,7]. APP, a glycoprotein composed of approximately 770 amino acids, is widely expressed in the cell membranes of various cell types, including neurons [8]. Disruptions in the normal processing of APP can lead to the formation of toxic oligopeptides composed of 39 to 43 amino acid residues, resulting in the assembly of protofibrils and fibrils, which subsequently aggregate into deposits visible under microscopic examination [9]. The formation of these deposits requires β-amyloid to maintain a stable structure; mutations that destabilize β-amyloid can prevent aggregation. Among the β-amyloid species, the (amyloid beta)Aβ-42 isoform produced via the aforementioned enzymatic pathways exhibits particularly potent cytotoxic effects, primarily against neurons. This toxicity is largely mediated by the generation of reactive oxygen species, which are harmful to neural cells [10,11]. Aβ-induced toxicity is further amplified by disruptions in calcium homeostasis, resulting from lipid membrane disturbances that ultimately lead to neuronal death [12-14]. Another critical element in plaque formation is the Tau protein, which facilitates the assembly of tubulin into microtubules. Microtubules form the structural framework of intracellular transport pathways and are essential for processes such as cell division, where they contribute to mitotic spindle formation. In AD, the pathological accumulation of neurofibrillary tangles results from the hyperphosphorylation of the Tau protein [15]. The neurotoxicity associated with Tau arises through two main mechanisms: either the loss of its normal function, leading to microtubule destabilization, or a toxic gain-of-function that promotes neuronal apoptosis [16]. Numerous studies have demonstrated a link between β-amyloid accumulation and Tau aggregation, underscoring their combined role in the final stages of Alzheimer’s disease pathogenesis [17].

The brain consumes approximately 20% more oxygen compared to other organs, rendering it particularly vulnerable to damage from reactive oxygen species (ROS) and reactive nitrogen species (RNS) [18,19]. These species are highly reactive and unstable due to the presence of unpaired electrons within their molecular structures. In individuals diagnosed with Alzheimer’s disease, oxidative damage and associated neuronal tissue alterations are frequently documented [20,21]. Neurons, rich in polyunsaturated fatty acids, are especially susceptible to interactions with ROS and RNS. These interactions promote lipid peroxidation, alter the redox states of metal ions bound to β-amyloid, and impair mitochondrial function, all of which contribute to neuronal apoptosis. Furthermore, oxidative stress-induced lipid peroxidation and deoxyribonucleic acid (DNA) strand breaks accelerate neuronal aging and cell death.

Neuronal apoptosis is closely linked to the development of cortical atrophy, a pathological process that underlies the progression of AD [22]. Lipid peroxidation of polyunsaturated fatty acids leads to the production and accumulation of reactive aldehydes, including 4-hydroxy-2,3-nonenal (HNE), malondialdehyde, and F2-isoprostanes. These lipid peroxidation by-products have been shown to promote Tau hyperphosphorylation and disrupt calcium homeostasis at the neuronal membrane, thereby initiating apoptotic cascades within neurons [23]. In addition to lipid damage, oxidative stress exerts deleterious effects on both nuclear and mitochondrial DNA, predominantly through ROS generated within the brain [24]. This results in the fragmentation of DNA strands and disruption of DNA-protein interactions. Extensive research has demonstrated that oxidative modifications to DNA nucleotides produce characteristic lesions, which serve as reliable biomarkers for evaluating the extent of oxidative injury [24-26]. Another critical mechanism by which oxidative stress contributes to AD pathogenesis involves the reaction of ROS with glycoproteins, leading to the formation of advanced glycation end products (AGEs). These AGEs possess significant neurotoxic properties and can trigger the release of inflammatory cytokines, such as interleukin-1 (IL-1) and tumor necrosis factor-alpha (TNF-α), thereby promoting a sustained neuroinflammatory state in the Alzheimer’s brain [27]. Notably, oxidative stress has also been implicated in the pathological conversion of physiological Tau into AGE-modified forms, which further propagate the molecular and cellular dysfunctions characteristic of AD.

The central nervous system (CNS) lacks a conventional lymphatic network. Instead, its two principal fluids, cerebrospinal fluid (CSF) and interstitial fluid, are cleared through distinct pathways. CSF predominantly drains into the venous system via arachnoid villi and granulations [28]. Enlarged perivascular spaces have emerged as critical imaging markers in conditions such as cerebral small vessel disease, multiple sclerosis, and even in the context of normal aging. However, the prevailing notion that there exists a true anatomical space surrounding cerebral vessels in the healthy brain is inaccurate. Histological investigations [29] and electron microscopy analyses [30] in human samples reveal the absence of a true empty space; rather, a compartment filled with extracellular matrix is located adjacent to the vessel wall. This compartment results from the fusion of the basement membranes of astrocytic end feet with those of the leptomeningeal adventitia. It is what is commonly referred to as the perivascular space.

In vivo rodent studies have demonstrated that this perivascular compartment facilitates the entry and convective movement of CSF into the brain, a mechanism often referred to as glymphatic influx [31]. Meanwhile, interstitial fluid exits the brain parenchyma by traveling along the basement membranes of CNS capillaries, arterioles, and arteries, a route known as intramural periarterial drainage (IPAD) [31-33]. Impairment of IPAD is implicated in the deposition of amyloid-β (Aβ) within these basement membranes, leading to cerebral amyloid angiopathy (CAA) [34].

Distinctively, IPAD operates within basement membranes and in a direction counter to that of arterial blood flow. Ultrastructural research has shown that when tagged Aβ is introduced into hippocampal tissue, it infiltrates capillary walls and moves along the basement membranes surrounding the smooth muscle cells of arterioles and arteries within the tunica media [35]. The motility of IPAD is driven by the rhythmic contraction and relaxation of vascular smooth muscle cells, propelling interstitial fluid and solutes against the direction of the blood stream [36].

As CAA advances, it induces numerous pathological changes in the vasculature, including compromised smooth muscle function, thickening of vessel walls, loss of autoregulatory capacity, and occasional vessel rupture. Enhancing the efficiency of IPAD might represent a therapeutic avenue to facilitate Aβ clearance and mitigate its accumulation. Observations that CAA progression worsens following Aβ immunotherapy, with Aβ42 being found within IPAD pathways, suggest that solubilized Aβ42 may be released from plaques but subsequently becomes trapped within these drainage routes [37].

## Material and Methods

The surgical procedures outlined in this chapter require the use of various materials and reagents. Certain reagents must be prepared in advance, including the injection system, anesthesia induction chamber, anesthetic agents, and the StereoDrive stereotaxic system with a drill and microinjection unit. All experiments were conducted in accordance with national and international guidelines for the care and use of laboratory animals. This study is reported in compliance with the ARRIVE (Animal Research: Reporting of In Vivo Experiments) guidelines, ensuring transparency, reproducibility, and rigorous reporting of in vivo experimental research.

### Stereotaxic system

**Figure 1:**
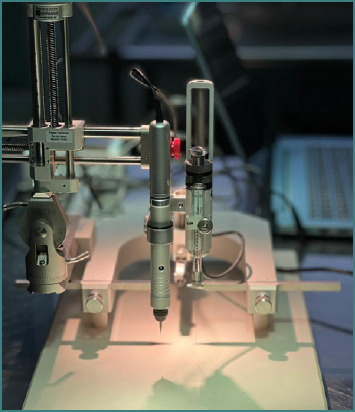
The stereotaxic robot

The StereoDrive system comprises several key components. The stereotaxic robot represented in Figure 1 represents the central mechanical element of the system. The drilling and microinjection unit is installed on the mediolateral axis of the stereotaxic frame, with both the drill and syringe motor-driven. Communication between the computer running the StereoDrive software and the stereotaxic machine is handled by a controller that translates software-generated instructions into motor commands for the stereotaxic robot. There are several axes for controlling the injection, probe positioning, and displacement, which are governed by movement along the dorsoventral (DV), mediolateral (ML), and anteroposterior (AP) axes. Any adjustment along one of these axes results in a corresponding change in probe location. Each axis is motorized, enabling accurate, computer-controlled positioning. It is essential to remain within the defined travel limits for each axis, as exceeding these limits may damage the equipment.

**Figure 2:**
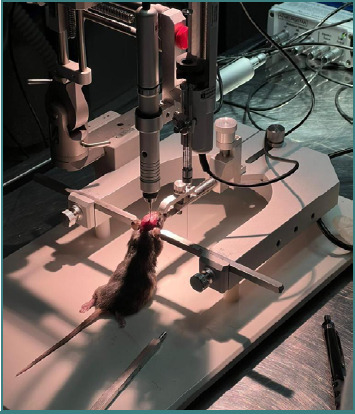
The mice were placed in the stereotaxic machine

The mice are immobilized within the stereotaxic frame during surgery using ear bars, a nose clamp, and a mouth bar. The ear bars prevent lateral displacement of the head, while the nose clamp and mouth bar restrict vertical movement. The animal should be positioned with the tail oriented toward the open end of the frame and secured using all fixation components represented in Figure 2. Achieving a perfectly horizontal head position is rarely feasible. Therefore, the head should be aligned as evenly as possible. Any residual tilt can be compensated for by adjusting the stereotaxic atlas using the software’s atlas alignment function.

The drill-and-injection robotic module integrates two functional components, a drilling mechanism and an injection system, into a single unit. This combined device enables surgical procedures to be carried out without the need to replace instruments on the stereotaxic frame.

### Procedure of injection in mice

#### Drill and Syringe Attachment

The first phase of this procedure is attaching the drill and syringe to their positions and synchronizing them. The next step is to load the syringe (Hamilton 5 microliters type) with the substance needed for the experiment.

#### Induction of general anesthesia

The oxygen flow rate is first adjusted to 1.7 L/min, after which the airflow valve is set to deliver the 5% isoflurane-oxygen mixture to the induction chamber. The mouse is weighed, then placed in the chamber, and anesthesia is initiated by setting the isoflurane vaporizer to level 4. The depth of anesthesia is continuously assessed using the pedal withdrawal reflex, with the surgical plane defined by the absence of a response to toe pinch. Once adequate anesthesia is achieved, the airflow valve is switched to redirect the oxygen isoflurane mixture to the stereotaxic frame. The animal is then carefully transferred from the induction chamber to the isoflurane mask attached to the stereotaxic machine. A longitudinal incision between the frontal and occipital regions is performed, and retractors are used for exposure of the bone.

#### Atlas adjustment

The atlas adjustment panel is opened, and the option for tilt and scaling correction is selected. The syringe is then advanced to the lambda landmark until it makes gentle contact with the skull surface, after which the lambda position is confirmed using the corresponding command. The bregma reference is assumed to have been previously defined during the drill-and-syringe synchronization process. Next, the syringe is displaced 2 mm to the left. From this position, it is lowered along the DV axis until it lightly contacts the skull, and the left reference point is recorded. The syringe is subsequently moved 2 mm to the right, lowered again in the DV direction to touch the skull, and the right reference point is set. Once these adjustments are completed, the tilt correction interface is closed.

#### Target selection

Planning mode is first activated by selecting the corresponding command. The anteroposterior slices of the atlas are then browsed using the mouse scroll wheel, and the desired target location is defined by clicking directly on the atlas image. The selected target is saved using the storage function, after which the interface returns to standard operating mode.

#### Drill and injection

The drilling procedure is initiated by opening the drill interface from the tools menu and selecting the drilling option. The drill position is then defined by selecting the previously stored target, which can be visually verified using the display function. Once confirmed, the drill is moved to the designated entry point, maintaining a predefined safety distance above the skull surface. An appropriate step size for DV advancement is selected, and drilling is initiated by advancing the drill in incremental steps. When the drill bit makes contact with the skull, typically indicated by a change in acoustic feedback, the DV movement is immediately halted. The skull surface position is then recorded, and drilling is continued with adjusted advancement parameters until the dura mater is reached. The autostop function ends the procedure automatically upon detection of cerebrospinal fluid.

The syringe is first selected as the active instrument using the corresponding control. The needle is then aligned with the previously defined target by selecting the stored coordinates and navigating to the entry point, ensuring that the syringe is positioned at a predefined safety distance above the target. The injection control interface is subsequently opened, and the controlled injection mode is initiated. The required injection parameters, including volume and delivery rate or duration, are then specified. Following parameter confirmation, the syringe needle is advanced to the target location by reselecting the target coordinates and executing the positioning command. Injection is initiated using the injection control, after which the needle is withdrawn in the DV direction using either relative movement or a direct repositioning command.

This approach allows the administration of very small volumes of tracers into the mouse brain parenchyma at a controlled infusion rate that is within the physiological range (0.5 μL delivered over 2.5 minutes). After finishing the injection, the capillary tip is kept in place for 2 minutes to allow local diffusion of the injected solution and to minimize backflow along the injection track. Subsequently, an additional 5 minutes is allowed to facilitate tracer distribution and perivascular drainage.

## Results

### Procedural performance and reproducibility

The described workflow enabled consistent intrahippocampal targeting using motorized stereotaxic navigation with atlas tilt correction. Synchronization of the drill and syringe units facilitated seamless transitions between drilling and microinjection without instrument exchange, thereby reducing handling-related variability. The stepwise DV advancement and surface registration improved the reproducibility of skull reference acquisition and entry hole positioning.

### Intraoperative stability and targeting accuracy

Head fixation using ear bars, a nose clamp, and a mouth bar provided stable immobilization throughout the procedure. Minor residual head tilt, unavoidable in most animals, was effectively compensated by the software-based atlas adjustment. Target selection in planning mode and subsequent “go-to entry” positioning reproducibly placed the drill and syringe above the intended coordinates at a safety distance before DV advancement, supporting an accurate approach to the target region.

### Controlled tracer delivery and post-injection handling

Microinjections were performed at a controlled infusion rate consistent with physiologically relevant delivery (0.5 μL over 2.5 min). Maintaining the capillary tip in situ for an additional 2 min after infusion minimized reflux along the needle track and promoted local diffusion of the tracer solution. The subsequent 5 min dwell period allowed the tracer to engage perivascular pathways under stable conditions before needle retraction.

### Practical considerations and procedural endpoints

A clear acoustic change was consistently observed upon initial drill contact with the skull surface, enabling immediate cessation of DV movement and reliable surface setting. Progressive drilling with adjusted step parameters allowed controlled advancement toward the dura mater. The autostop function provided an additional safety mechanism by terminating drilling upon detection of cerebrospinal fluid, reducing the risk of over penetration.

## Discussion

### Methodological value for studying perivascular clearance in Alzheimer’s disease

Intramural periarterial drainage is increasingly recognized as a key route for interstitial solute clearance, and its impairment has been implicated in amyloid-β retention within vascular basement membranes and the development of cerebral amyloid angiopathy. The present protocol addresses a central technical barrier in this research area: the ability to deliver very small tracer volumes to defined hippocampal sites with high spatial precision while preserving local microvascular and extracellular matrix integrity. By combining controlled infusion with standardized post-injection dwell times, the method is well-suited to experiments aimed at tracking early tracer redistribution and clearance along perivascular pathways.

### Importance of controlled infusion and dwell periods

The injection parameters and the deliberate post-injection holding periods serve two critical purposes. First, they reduce pressure-driven artefacts, including parenchymal tearing, local edema, and nonphysiological bulk flow, which can confound the interpretation of perivascular transport. Second, leaving the capillary tip in place after injection mitigates backflow, which is a common source of false positive 'spread' along the needle track. The additional waiting interval before withdrawal provides a practical window for early tracer engagement with perivascular routes, supporting downstream imaging or histological quantification.

### Advantages of motorized stereotaxy and atlas tilt correction

A recurring limitation in stereotaxic microinjection studies is the variability introduced by subtle differences in skull positioning and reference-point acquisition. The software-based correction for tilt and scaling, implemented through lambda confirmation and bilateral DV reference points, improves alignment between the atlas and the individual animal. The motorized DV/ML/AP axes further enhance precision and reduce operator-dependent variability compared with purely manual approaches. These elements are particularly relevant for hippocampal injections, where small coordinate deviations can shift delivery between closely adjacent structures (CA1/CA3/dentate gyrus), potentially altering tracer kinetics and interpretation.

### Technical difficulty and sources of variability

Despite these advantages, intrahippocampal injection remains technically demanding. Variability can arise from skull thickness, minor deviations in drilling angle, differences in dura mater resistance, and individual anatomical variation. Inadequate fixation, imperfect leveling, or premature needle withdrawal may increase reflux or off-target delivery. The protocol therefore benefits from explicit checkpoints (surface setting, safety distance positioning, stepwise DV advancement) and from structured operator training to ensure consistent execution. In method-focused studies, documenting procedural failures, off-target placements, and exclusion criteria is essential to strengthen reproducibility and transparency.

### Limitations and recommended reporting for future work

As described, the manuscript focuses on the surgical and stereotaxic workflow. However, the interpretability of IPAD-related findings ultimately depends on rigorous downstream validation. Future implementations should report the proportion of injections confirmed within the intended hippocampal subregion, quantitative measures of reflux or track-associated spread, and tracer detection along vascular basement membrane-associated compartments at defined post-injection time points. Including representative images of injection sites and perivascular tracer localization, along with a brief description of histological processing and analysis pipelines, would further strengthen the method’s translational relevance.

### Implications for neuroscience applications

Overall, this protocol provides a technically rigorous platform for experimental interrogation of perivascular clearance mechanisms in vivo. Given the emerging evidence linking cerebrovascular dysfunction, impaired clearance, and amyloid accumulation in Alzheimer’s disease, standardized and reproducible intrahippocampal microinjection methods are valuable for mechanistic studies and for evaluating interventions aimed at improving vascular smooth muscle function and solute elimination.

## Conclusion

The intrahippocampal injection protocol presented here is a reliable, highly standardized method for delivering extremely small tracer volumes into the mouse brain parenchyma. The integration of a fully motorized stereotaxic platform with synchronized drilling and microinjection functions enables precise anatomical targeting, consistent injection depth, and reproducible infusion parameters, while simultaneously reducing procedural variability and minimizing mechanical trauma to the surrounding tissue.

The implementation of a controlled, physiologically relevant infusion rate, together with defined postinjection dwell periods, enables reproducible evaluation of tracer dispersion within the brain parenchyma and subsequent intramural periarterial drainage. This level of temporal and spatial control is essential for accurately characterizing perivascular clearance mechanisms and detecting subtle alterations in fluid transport characteristic of neurodegenerative conditions such as AD.

Although technically demanding, the procedure offers high accuracy and adaptability, making it suitable for investigating subtle alterations in perivascular transport mechanisms. When performed with careful atlas alignment and strict adherence to stereotaxic coordinates, this method represents a powerful experimental tool for exploring cerebrovascular clearance pathways and their dysfunction in experimental models of AD.
